# PP60 Enhancing Patient Empowerment: European Capacity Building Initiative And Curriculum Development In Health Technology Assessment Training

**DOI:** 10.1017/S0266462324002319

**Published:** 2025-01-07

**Authors:** Milena Muehler, Silke Siebert, Petra Schnell-Inderst, Sonja Roßmann, Sibylle Puntscher, Tanja Planinschitz, Lára R. Hallsson, François Houÿez, Julien Delaye, Valentina Strammiello, Uwe Siebert

## Abstract

**Introduction:**

The new Regulation (EU) 2021/2282 on health technology assessment (HTAR) highlights the increasing importance of patient perspectives, emphasizing their crucial role in HTA. The European Capacity Building for Patients (EUCAPA) project aims to empower patients for active involvement in HTA. EUCAPA focuses on equipping patients with capacities through the development of HTA training, including introductory, fast-track, and extended training programs.

**Methods:**

Within the EU-funded EU4Health program, the EUCAPA consortium was tasked with capacity building for patients and patient representatives. Training programs were developed using a coproduction approach, integrating patient representatives’ perspectives into both curriculum development and the design of training sessions. This innovative approach involved close collaboration between patient representatives and HTA scientists, jointly shaping the program content. The curriculum was designed to incorporate the heterogeneous medical and cultural backgrounds and varying levels of prior knowledge among participants, recognizing diverse experiences that individuals bring to the training. To enhance accessibility, a blended learning approach with offline, virtual, and in-person parts was adopted.

**Results:**

The training comprised introductory (two hours), fast-track (eight hours), and extended (four days) training sessions. Training modules include competencies such as a profound understanding of HTA processes and key principles, domains, impact of HTA, outcome measurements, and study designs to raise awareness, promote, and enhance patient engagement as foreseen in the HTAR. The training modules focus on empowering patients to advocate for their rights and communities and to contribute meaningfully to comprehensive treatment evaluation. Specifically, modules on communication and personal skills, patient involvement, and engagement at both national and European levels underscore promotion of patient empowerment. Training sessions can be accessed online (EUCAPA.eu).

**Conclusions:**

The training development within the EUCAPA project addresses the evolving landscape of joint HTA by emphasizing the significance of patient perspectives, fostering core competencies, and responding to the increasing demand for active patient involvement and advocacy at both national and supranational levels.

